# Online recognition and yield estimation of tomato in plant factory based on YOLOv3

**DOI:** 10.1038/s41598-022-12732-1

**Published:** 2022-05-23

**Authors:** Xinfa Wang, Zubko Vladislav, Onychko Viktor, Zhenwei Wu, Mingfu Zhao

**Affiliations:** 1 Sumy National Agrarian University, Sumy, Ukraine; 2 Henan Institute of Science and Technology, Xinxiang, Henan China

**Keywords:** Electrical and electronic engineering, Image processing, Computer modelling

## Abstract

In order to realize the intelligent online yield estimation of tomato in the plant factory with artificial lighting (PFAL), a recognition method of tomato red fruit and green fruit based on improved yolov3 deep learning model was proposed to count and estimate tomato fruit yield under natural growth state. According to the planting environment and facility conditions of tomato plants, a computer vision system for fruit counting and yield estimation was designed and the new position loss function was based on the generalized intersection over union (GIoU), which improved the traditional YOLO algorithm loss function. Meanwhile, the scale invariant feature could promote the description precision of the different shapes of fruits. Based on the construction and labeling of the sample image data, the K-means clustering algorithm was used to obtain nine prior boxes of different specifications which were assigned according to the hierarchical level of the feature map. The experimental results of model training and evaluation showed that the mean average precision (mAP) of the improved detection model reached 99.3%, which was 2.7% higher than that of the traditional YOLOv3 model, and the processing time for a single image declined to 15 ms. Moreover, the improved YOLOv3 model had better identification effects for dense and shaded fruits. The research results can provide yield estimation methods and technical support for the research and development of intelligent control system for planting fruits and vegetables in plant factories, greenhouses and fields.

## Introduction

Dwarf fruit and vegetable varieties are most suitable for soilless cultivation on the planting layer shelf, and will become the first choice for agricultural production in plant factories with artificial lighting (PFALs)^1–4^. Fruit counting and yield estimation are the important basis for planning plant factory planting planning and marketing strategy, and also an important part of plant factory information service system data^5–7^. Through the real-time statistics and prediction of tomato fruit time series yield information, and the corresponding production control, in order to achieve the accurate response of supply orders, it is of great significance to solve the current tomato production capacity fluctuations, production process discontinuity and other problems^8,9^. The visual information acquisition of tomato fruit is an important prerequisite to support intelligent yield estimation. However, the tomato plants in the plant factory are clustered and disordered, and the stems, leaves and fruits grow densely and overlap with each other, which makes the fruit image feature recognition become an important factor limiting the accurate estimation of tomato yield.

In view of the unstructured features of the appearance, posture and size of crop objects, it is difficult to realize the accurate recognition of image features based on single threshold classification method. By fusing multiple information such as color, shape, texture and pose to establish an adaptive classification and recognition model, it is an effective way to realize the recognition of complex features^10^. The deep learning model^11,12^ centered on the multi-layer convolution feature extraction network avoids the complex process of traditional machine learning model construction, has higher recognition accuracy, and has unique advantages for the perceptual fusion of multi-visual information of agricultural work objects^13–17^. Wang et al.^18^ used fuzzy C-means clustering algorithm to segment tomato red fruit, fruit stalk and leaf images, and the recognition accuracy of mature tomato pixels reached 83.45%. Ma et al.^19^ used target recognition methods based on the dense and sparse reconstruction (DSR) method and circular random Hough transformation to detect immature tomato fruit images with a correct recognition rate of 77.6%. Sun et al.^20^ proposed a broccoli seedling detection method based on Faser R-CNN in a natural environment, with an average accuracy of 91%. Muresan et al. proposed an optimization method based on deep convolution network structure to classify and identify eight types of fruits with an accuracy of more than 95%^21^. Cui et al. used the visualization method to compare the feature extraction differences of six types of convolution neural networks with different depths, determined the best convolution network Alexnet^22^ and Vgg16^23^, and the recognition accuracy can reach more than 93%^24^. Williams et al. proposed a deep learning based singular fruit recognition method and applied it to the detection of dense fruits by harvesting robots with an accuracy of 76.3%^25^. Zhao et al. proposed a method for locating apples based on YOLOv3 deep convolution neural network, with an accuracy of 97%^26^. The above target recognition algorithms are mainly focused on specific color targets, however, tomato yield estimation during natural growth requires identification of green and red fruits of different maturity levels.

In order to accurately predict fruit and vegetable yields in plant factories, the dynamic recognition methods of tomato red and green fruits were studied, and the recognition accuracy of dense tomatoes in interwoven plexus plants was improved through the improved YOLOv3 deep learning model. The results can provide the methods of estimating production and technical support for the research and development of tomato production intelligent control system.

## Materials and methods

### Planting and growth environment of dwarf tomato in PFAL

In the enclosed space, the temperature and humidity of planting space in PFAL are usually controlled by air conditioning and dehumidifier, and artificial light illumination system and multi-layer layered soilless cultivation techniques are used to achieve the purpose of Industrial Planting. In Europe, North America and other regions, it is often called stereo planting system or vertical farm^27–32^. In order to make full use of the space and expand the planting area, the height of a layer shelf is limited, so the crops planted are more leaf vegetables and dwarf eggplant fruits.

**Figure 1:**
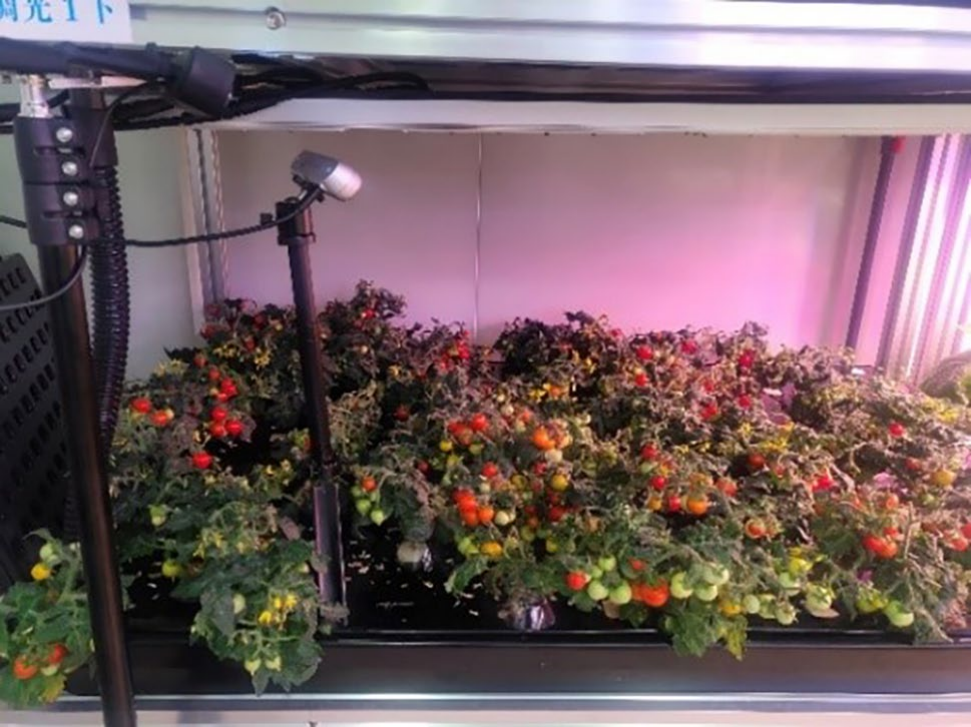
Micro-Tom dwarf tomatoes planted in the PFAL laboratory of our university and its image acquisition system.

The experiment was carried out in the laboratory of PFAL of Henan Institute of science and technology from January 2021 to August 2021. The tomato material used in the experiment is dwarf Micro-Tom tomato variety, and the seeds are provided by the teachers engaged in botany and cultivation research in our project team. The tomato seedlings begin to blossom and bear fruit about 25 days after transplanting and planting, and the flowering and bearing can last for several months. In order to detect tomato fruit in real time, Intel Realsense D455 RGB-D camera and iDS-TCV441-CF industrial camera system is used to collect tomato images and video data, shown in Fig. 1 for details. The total height of planting shelf generally depends on the spatial structure, which is about 3000–5000 mm high. The top layer is about 1000 mm away from the ceiling, and the first layer is 500–700 mm away from the ground. The height of a planting layer is generally 600–800 mm, and there are usually 2–5 layers. The highest growth height of dwarf tomato was 300–500 mm, and the fruit bearing area at the height of 200–500 mm was mainly collected for yield estimation. The intelligent yield estimation equipment moves on the track between the rows of plants, and its vision system obtains the image information of tomato plants on both sides in real time. (The authors declare that our plant experimental research and field research comply with relevant institutional, national and international norms and legislation and the pictures collected, and all of the plant samples used in our experiments were obtained in the plant factory and greenhouse laboratory of our university, and no wild plants or other protected plant species were used).

### Image photographing and acquisition system

In the plant factory, the computer vision system is the basis of fruit recognition and yield estimation under the natural growth conditions of plants, which is composed of a binocular vision camera or camera, portable computing unit, 5G communication module, guide rail fixed on the planting rack, pan tilt, other mechanical components, etc. In this experiment, the iDS-TCV441-CF industrial camera system produced in HKVISION of Canada was selected, with dual 4 megapixel lens, 800 mm away from the tomato plant, and the length and width of the field of view were 800 mm and 600 mm, respectively. The portable computing unit and 5G communication module are optional, built-in and fixed in the box connected with the guide rail, which is not used in this experiment. The pan tilt can be rotated vertically and horizontally to adjust the spatial attitude of the camera and collect images of different areas of tomato plants from different perspectives. The portable computing unit, 5G communication module and pan tilt can swim along the guide rail to capture tomato plants in different areas of the planting shelves.

### Image acquisition methods

**Figure 2:**
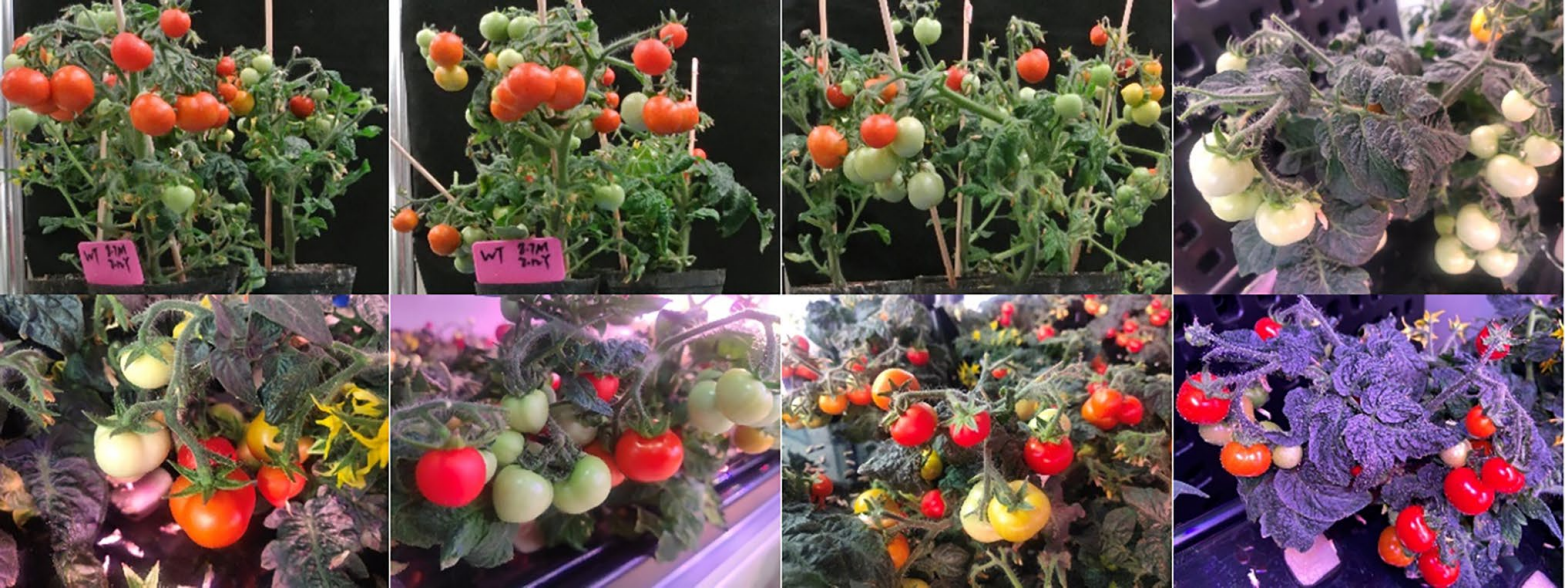
Samples of collected original image data.

In order to improve the accuracy of online yield estimation, in this experiment, we collected image data of substrate potted and artificial-lighting-hydroponic Micro-Tom tomato, respectively. When collecting potted tomatoes, we first took pictures of each plant in a horizontal view, fixed the focal length, shutter speed, aperture size and camera position, and took one picture of each plant rotating at a 60° angle. Then, it is the same as the horizontal front view shooting method, taking a 45° angle from the horizontal, fixing the focal length, shutter speed, aperture size and camera position, and taking one picture every 60°. Finally, one picture was taken vertically from the horizontal plane, and a total of 13 pictures were taken for each plant. In addition, it is to take photos in clusters, putting the three plants together in a compact way, and the photo taking method is the same as that of a single plant. In this study, 120 tomato plants were photographed in three times, and a total of 1560 single plants and 780 pictures of tomato fruit bearing plants three clusters were obtained. During the image acquisition of artificial-light-hydroponic Micro-Tom tomato, we took random photos at different times of the day, from different angles and with different camera parameters, and obtained a large number of pictures. We selected 2600 pictures for fruit data labeling and labeled 4000 tomato fruits for model training. In this way, we obtain larger original data, enrich the data set for model training, and increase the universality of the trained model. The sample of collected original image data are shown in Fig. 2.

### Image data enhancement

**Figure 3:**
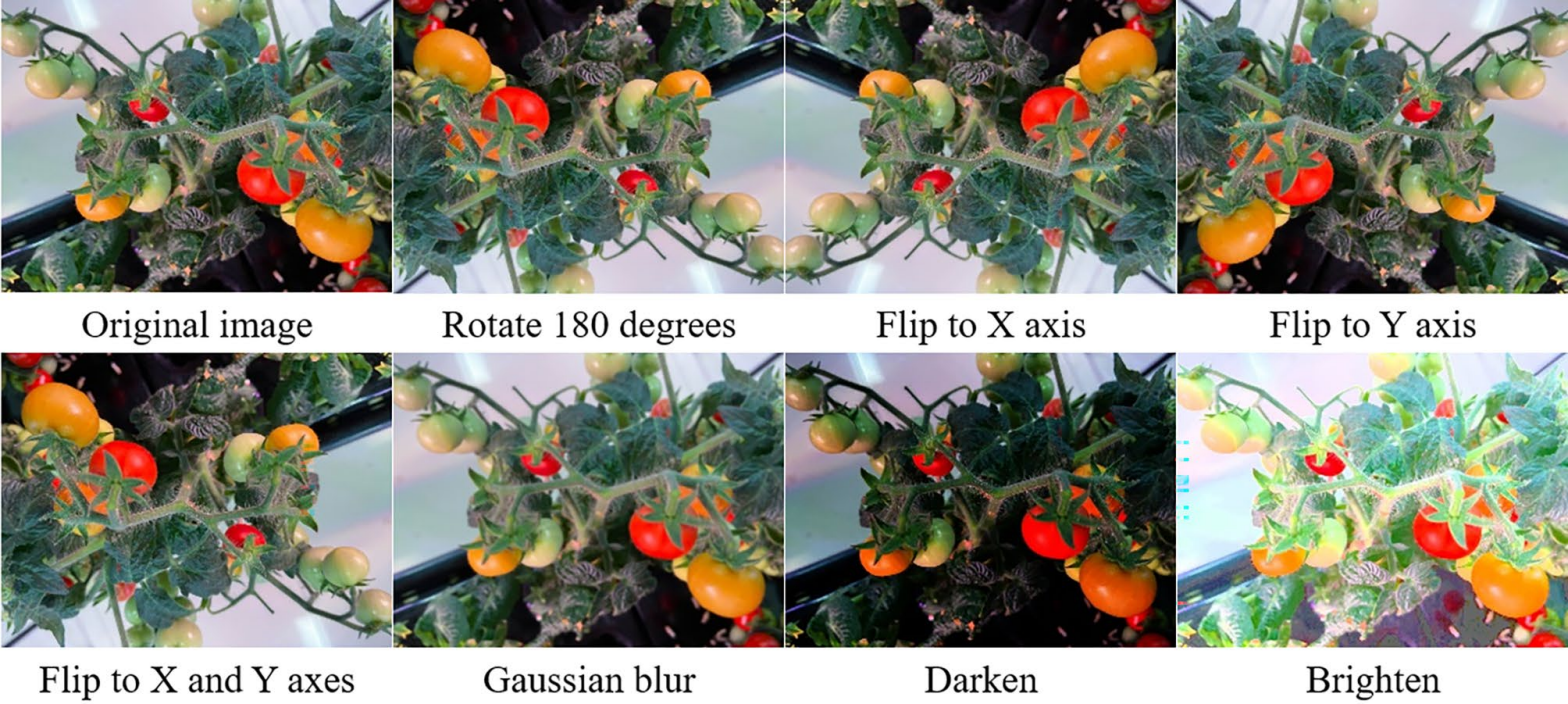
Enhanced illustration of the original image data.

In order to enrich the original image data and increase the universality and robustness of the data set, we processed the original image data with various algorithms such as rotation, flip, mirror image and blur, as shown in Fig. 3.

### Improved yolov3 recognition model

#### Principle of YOLO target recognition algorithm

**Figure 4:**
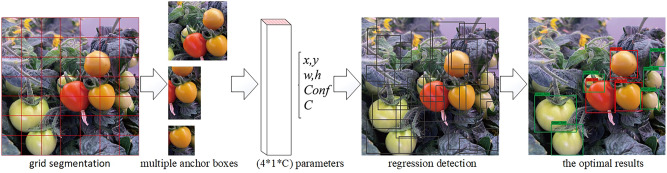
Principle of YOLO target recognition algorithm.

The basic principle of YOLO^33–35^ algorithm (shown in Fig. 4) is that the input picture is divided into *S* × *S* rasterized cells. If the detected target center falls into a specific cell, the cell is responsible for detecting the target, that is, the probability that itself has a target is $P_{obj} = 1$. It is preset that each cell produces *B* prior bounding boxes, and the intersection and union ratio between each bounding box and the real value bounding box is IOU, then the target location and category prediction in the image can be expressed by a tensor of $S \times S \times B \times \left( {4 + 1 + C} \right)$, in which 4 represents the coordinates of the prior bounding box (x, y), width and height $\left( {w,h} \right)$, 1 represents the confidence score, a total of 5 characteristic parameters, and $C$ represents the number of categories of the data set targets used. Through the training of continuous regression to the real boundary box, the location, confidence and category information of the final predicted target can be obtained. Finally, the best recognition result is screened by keeping the boundary box with the highest confidence coefficient.

#### Multi-scale feature extraction based on DarkNet53

**Figure 5:**
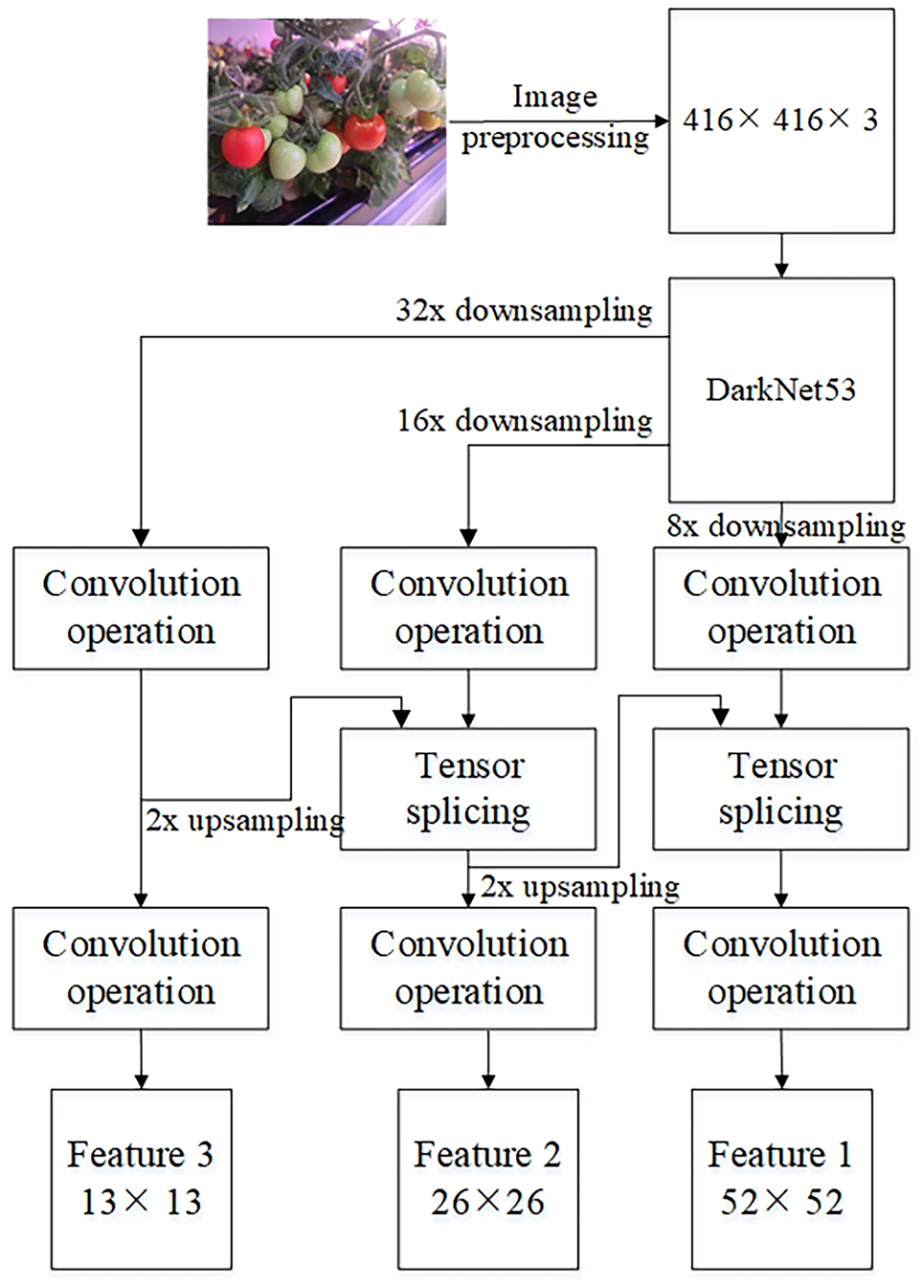
Principle of DarkNet53 Multi-scale feature extraction.

In the YOLOv3 algorithm, the DarkNet53 feature extraction network is used to obtain multi-scale image features, which overcomes the problem of missing detection of significant scale difference targets in the previous version of YOLO^36^. Before using the darknet53 feature extraction network, we need to preprocess the image data and adjust the image size to a unified image of 416 × 416 pixels. DarkNet53 takes 416 × 416 4 pixel image as input and undersamples 32 times, 16 times and 8 times, respectively, to obtain different levels of feature images, and then through up-sampling and tensor stitching, different levels of feature images are fused into feature maps with the same dimension. It contains multi-scale image features, which are helpful to improve the accuracy of the algorithm for small target detection. In view of the fact that red fruit and green fruit are detected in this paper, DarkNet53 feature extraction finally outputs three kinds of feature images with pixels of 13 × 13, 26 × 26, and 52 × 52, respectively, which are used as the basis for fruit target regression detection in the near and far field of view. Figure 5 shows the process and principle of multi-scale feature extraction based on darknet53.

#### Prior bounding box setting

According to the border marking information of the target sample, YOLOv3 sets a prior bounding box for regression detection in advance to improve the efficiency of target recognition. In this paper, using K-means clustering algorithm, using 1-IOU as the clustering index, nine prior bounding boxes with different specifications are obtained for three feature maps of different scales, and assigned according to the hierarchical level of the feature map, as shown in Table 1.

**Table 1:** Prior bounding boxes allocation of feature maps of different scales.

Feature map size/(pixels × pixels)	Prior bounding box size/(pixels × pixels)
13 × 13	(73 × 46), (93 × 75), (128 × 125)
26 × 26	(36 × 45), (52 × 34), (55 × 64)
52 × 52	(22 × 17), (25 × 31), (37 × 24)

Therefore, for the image of 416 pixels × 416pixels, after dividing the grid with 13 × 13, 26 × 26 and 52 × 52, respectively, three prior bounding boxes are set for each grid, and 13 × 13 × 3 + 26 × 26 × 3 + 52 × 52 × 3 = 10,647 predictions are needed to identify red fruit and green fruit.

#### Loss function and its improvement

The loss function of YOLO recognition algorithm includes three components: target location, confidence and classification, in which the target location loss defaults to the Euclidean distance between the target real bounding box center $\left( {\widehat{{x_{i} }}, \widehat{{y_{i} }}} \right)$, the width-height parameter $\left( {\widehat{{w_{i} }},\widehat{{h_{i} }}} \right)$ and the corresponding predicted bounding box parameters $\left( {x_{i} ,y_{i} } \right)$ and $\left( {w_{i} ,h_{i} } \right)$. The calculation method is shown in formula (1).

1 $${\text{Loss}}_{{{\text{word}}}} = \sum\limits_{{{\text{i}} = 0}}^{{{\text{S}}^{2} }} {\sum\limits_{{{\text{j}} = 0}}^{{\text{B}}} {1_{{{\text{ij}}}}^{{{\text{obj}}}} \left[ {\left( {x_{i} - \overset{\lower0.5em\hbox{$\smash{\scriptscriptstyle\frown}$}}{x}_{i} } \right)^{2} + \left( {{\text{y}}_{i} - \overset{\lower0.5em\hbox{$\smash{\scriptscriptstyle\frown}$}}{y}_{i} } \right)^{2} } \right]} } + \sum\limits_{{{\text{i}} = 0}}^{{{\text{S}}^{2} }} {\sum\limits_{{{\text{j}} = 0}}^{{\text{B}}} {1_{{{\text{ij}}}}^{{{\text{obj}}}} \left[ {\left( {\sqrt {w_{i} } - \sqrt {\overset{\lower0.5em\hbox{$\smash{\scriptscriptstyle\frown}$}}{w}_{i} } } \right)^{2} + \left( {\sqrt {h_{i} } - \sqrt {\overset{\lower0.5em\hbox{$\smash{\scriptscriptstyle\frown}$}}{h}_{i} } } \right)} \right]} }$$

In the equation, $LOSS_{word}$-target position loss function. $1_{ij}^{obj}$ indicates that the prior bounding box $j$ generated by cell $i$ contains the target, and $obj$ indicates that the object exists.

**Figure 6:**
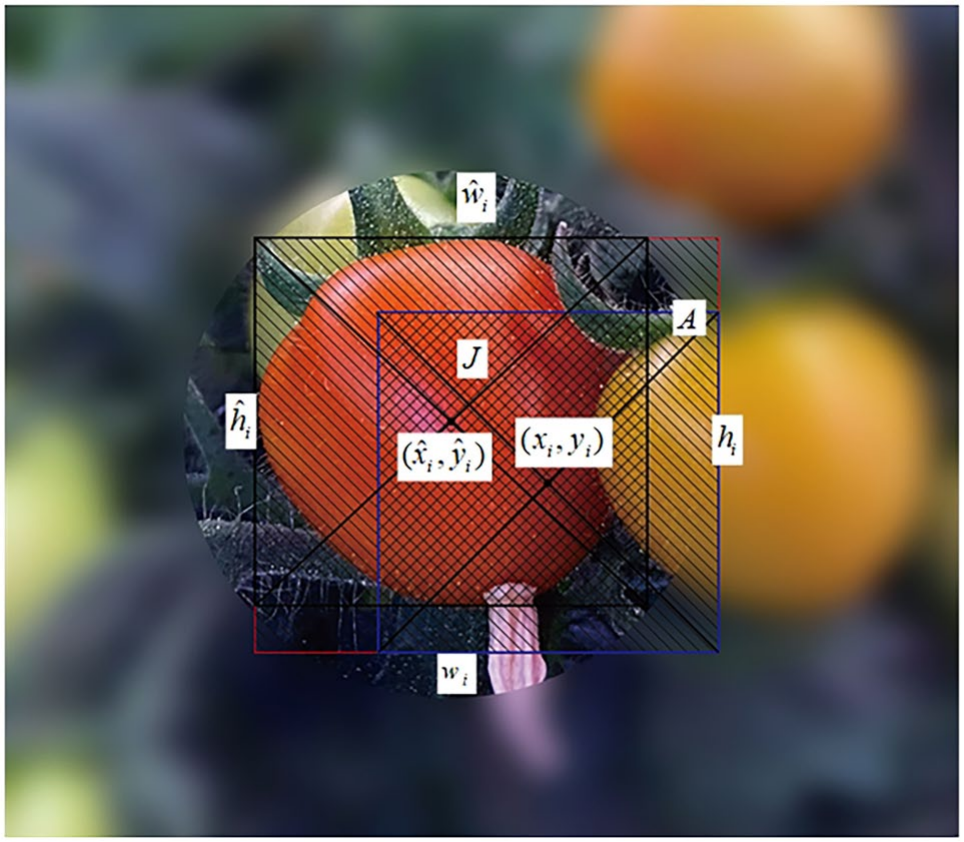
Tomato fruit border boxes GIOU.

In the process of image acquisition by the yield estimation vision system, the distance between the tomato and the camera changes dynamically, which makes the shape of the fruit show multi-scale changes in the image. If the Euclidean distance is used to evaluate the target bounding box deviation of tomato, the value of loss function is related to fruit size and does not have scale invariance, which is easy to cause the problem of missing detection of small fruits in the image. Therefore, the generalized intersection ratio^37,38^ (GIOU) parameter with scale invariance is used as the evaluation index of the deviation between the real bounding box and the predicted bounding box of the target. As shown in Fig. 6, the black box is the real fruit bounding box, the blue box is the prediction bounding box, the border intersection area is *J*, and the minimum surrounding bounding box (red) area is *A*, then the prior frame j of the target cell I and the GIOU_ij_ of the target real bounding box can be obtained by formula (2).

2 $$\left\{ {\begin{array}{*{20}l} {GIOU_{ij} = \frac{J}{U} - \frac{A - U}{A}} \hfill \\ {U = \hat{w}_{i} \times \hat{h}_{i} + w_{i} \times h_{i} - J} \hfill \\ \end{array} } \right.$$

When the prediction box coincides with the real box, GIOU takes a maximum value of 1. On the contrary, with the increase of the distance between them, GIOU tends to − 1. Accordingly, the target position loss function is improved to ${\text{Loss}}_{word} = \sum\limits_{i = 0}^{{S^{2} }} {\sum\limits_{j = 0}^{B} {1_{ij}^{obj} (1 - GIOU_{ij} )} }$, so that the greater the distance between the prediction box and the real box, the greater the loss value, and can overcome the influence of the target shape, and more accurately characterize the relationship between the frames, that is, it has scale invariance.

### Model training

#### Dateset construction

**Figure 7:**
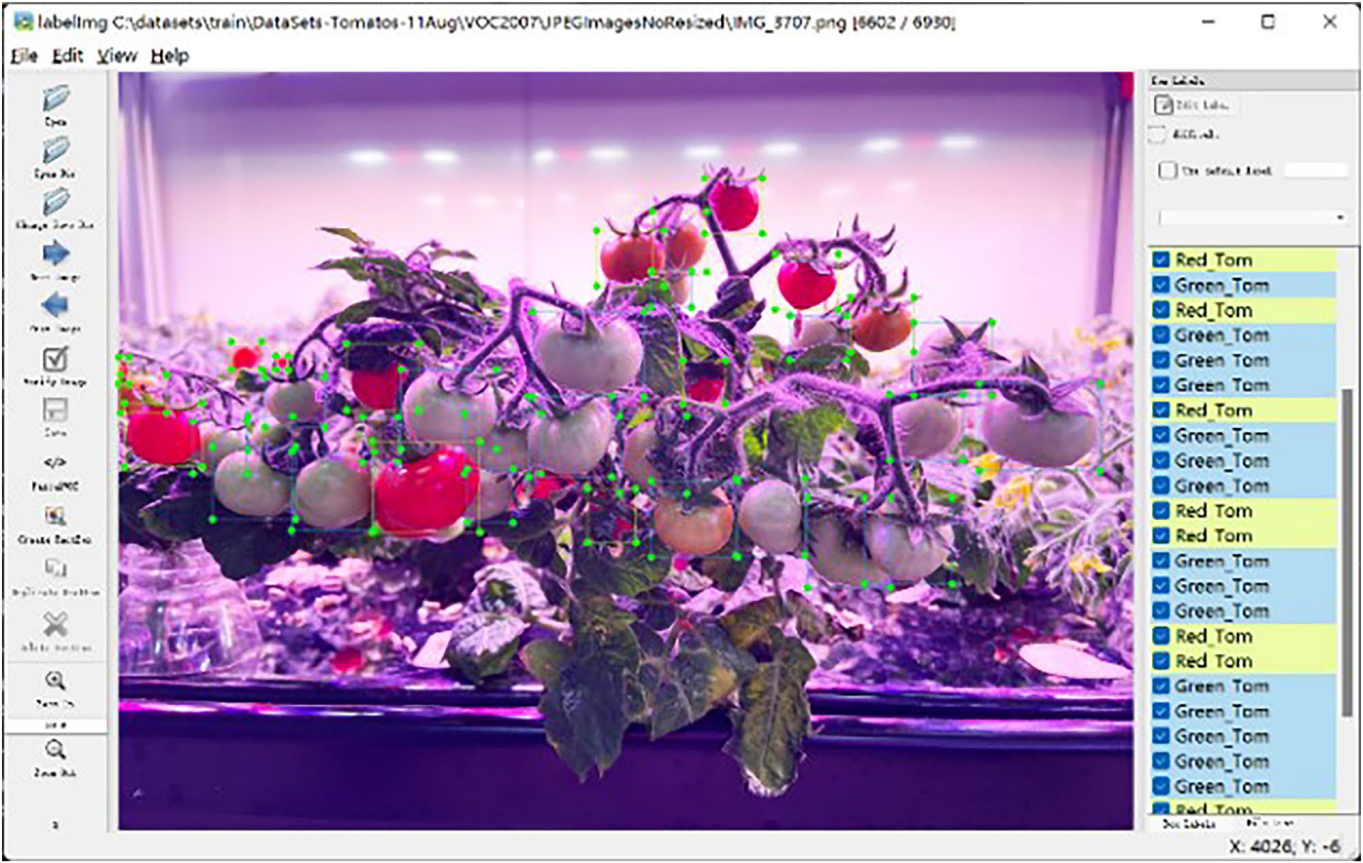
Data annotation demonstration.

We annotated a total of 3680 original images and 18,400 enhanced images, and constructed the basic data set and extended data set of Micro-Tom. Use the Labelimg annotation tool to label the areas of tomato red fruit and green fruit, and get the YOLO data set. Of the 4680 image samples, 1000 were randomly selected as the test sample set and the remaining 3680 as the training sample set. The data annotation process is shown in Fig. 7.

#### Algorithm running environment

The main hardware platform for running the algorithm is the TIANKUO I620-G30 server of SUGON, which is equipped with Intel Xeon E5-2680v4 processor, 128 GB of DDR4 2666 MHz memory, the motherboard using Intel C620 series chipsets and Nvidia GeForce RTX 1080TI. The software platform includes operating system CentOS 7.9, Python 3.8.8, pytorch deep learning framework 1.39, The CUDA 10.1 parallel computing framework was used with the CUDNN 7.6 deep neural network acceleration library, OpenCV computer vision 4.0.0, Matlab R2019a and other tools.

## Results and discussion

### Process and result of model training

**Figure 8:**
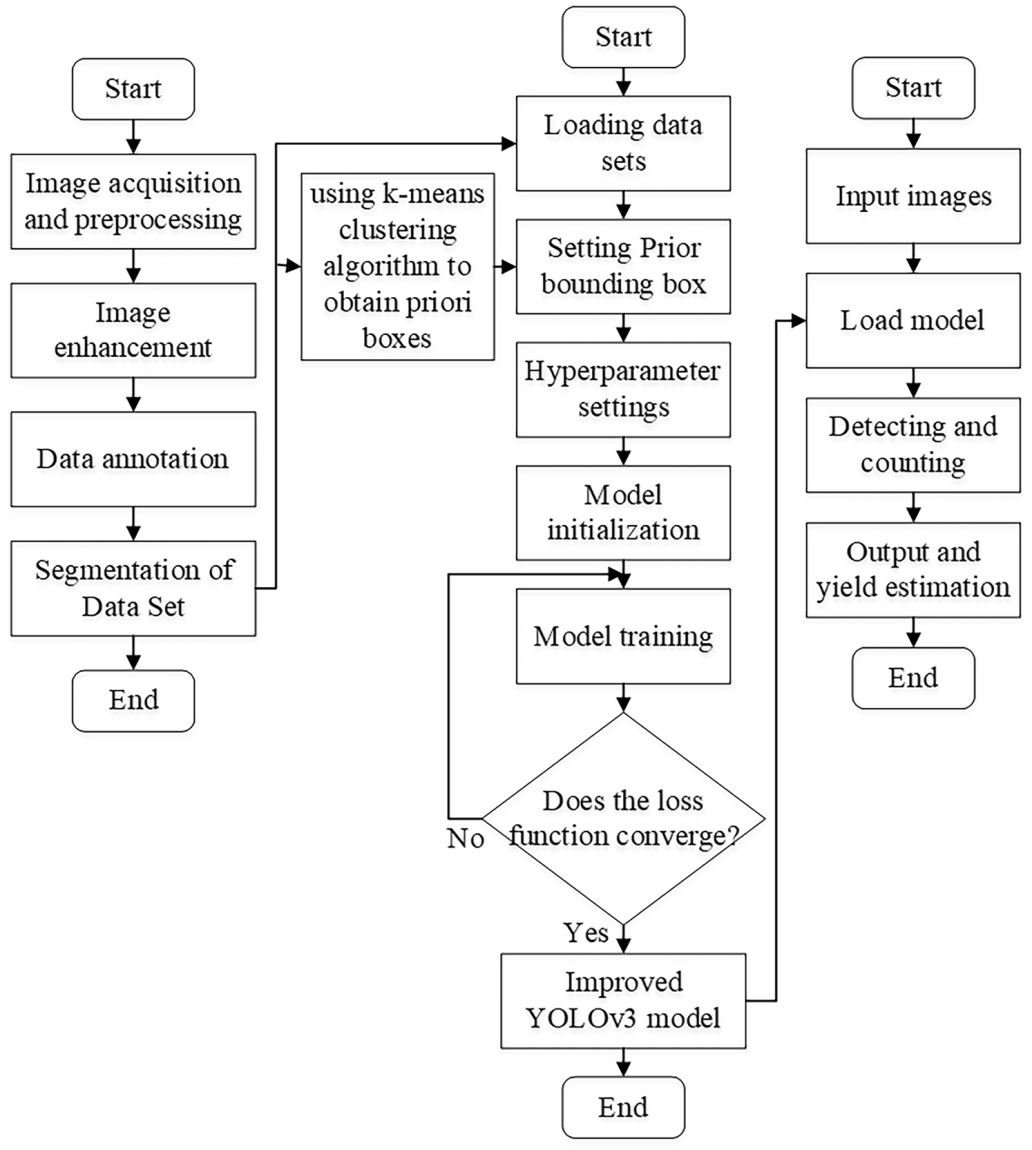
Algorithm flow chart.

Using the official weight parameters of YOLOv3, combined with the classification requirements of sample identification, to adjust the parameters of the output layer of the model. The model is trained based on the improved loss function, the training parameters are set as shown in Table 2, and the overall algorithm flow chart is shown in Fig. 8.

**Table 2:** Setting of model training parameters.

Parameters	Value
Iteration ordinal number	700
Batch size	8
Momentum parameter	0.9
Learning rate	0.001
Confidence threshold	0.5
Non-maximum suppression threshold	0.3

**Figure 9:**
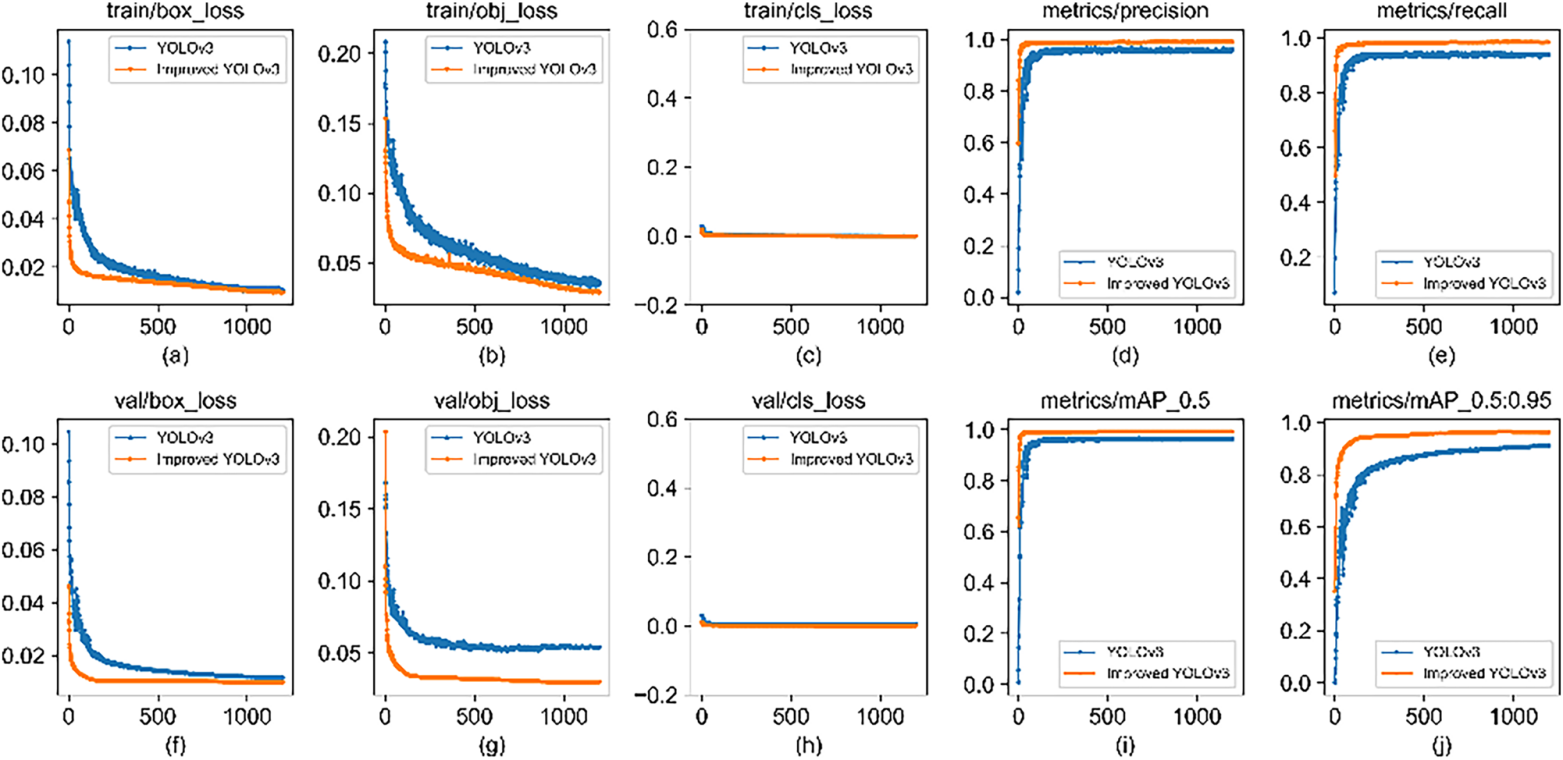
Loss function of training.

In the 700 iterative cycles, the change of the loss function is shown in Fig. 9. In the first 400 iterative cycles, the value of the loss function decreases obviously, and then decreases slowly. In order to ensure the convergence accuracy of the model, the learning rate is reduced after 400 iterative cycles. After 700 iterative cycles, the value of the loss function is dropped to 2 near with slight fluctuation, and it is considered that the model has reached stable convergence.

In the training process, the model is output every 10 iterative cycles, and the image of the test set is recognized and processed. Taking the mean average precision (mAP) as the evaluation index, the model with the highest accuracy is selected as the optimal model. The YOLOv3 of the same training process is compared with its improved model, as shown in Table 3. The mAP value of the traditional YOLOv3 is 96.5%, and that of the improved YOLOv3 is 99.3%, an increase of 2.8%, and the detection efficiency of the improved algorithm is basically the same as that of the traditional algorithm. The average detection time of a single image is 15 ms after loading the model.

**Table 3:** Performance comparison of algorithms.

Algorithm	mAP (Mean average precision %)	Single image detection time (ms)
YOLOv3	96.5	15
Improved YOLOv3	99.3	15

### Verification method

In order to verify the generalization performance of the model, field experiments were carried out in the plant factory laboratory of our university. 200 fields of view were randomly selected, and the tomato plant images were collected in real time by the yield estimation vision system, and the tomato fruit was counted and estimated by artificial, YOLOv3 and improved YOLOv3, respectively.

The experiment methods are as follows: (1) in the picking area, select different light and scale field of view, randomly collect images of tomato plants to ensure the diversity of data; (2) deliberately select some special image samples of sparse, dense and obscured fruits, and manually count the number of red and green fruits in the experimental images as control. (3) The YOLOv3 model and the improved YOLOv3 model are used for pattern recognition of the collected images and the fruits in the field of view are counted, and compared with the manual counting results to evaluate the accuracy of the yield estimation model.

## Discussion

Taking the manual counting results of red fruit and green fruit as reference, the recognition counting results of YOLOv3 and improved YOLOv3 were evaluated. The ratio of the identification and counting results of red fruit, green fruit and total fruit of the two models to manual counting is taken as the yield estimation accuracy of red fruit, green fruit and total fruit, respectively. The statistical results are shown in Table 4. The improved YOLOv3 model significantly improves the accuracy of tomato yield estimation, in which the accuracy of red fruit, green fruit and total yield estimation is 99.4%, 99.3% and 99.3%, respectively. Compared with the traditional YOLOv3 algorithm, the recognition accuracy is improved by 2.5%, 4.3% and 3.3%, respectively. From the results, we found that the detection accuracy of green fruit is lower than that of red fruit, whether the improved yolov3 model or the unchanged yolov3 model. By comparing and analyzing a large number of manually labeled image data and predicted result image data, it is found that there are occasional green areas very similar to green fruits in the image, which are incorrectly detected as tomatoes. In other images, tomatoes that are very close to the surrounding background are not detected correctly due to the influence of light. Therefore, the reason should be that the similarity between green fruit and plant background and the complexity of PFAL lighting jointly affect the detection accuracy of green fruit.

**Table 4:** Statistics of accuracy of tomato fruit yield estimation.

Model/algorithm	For red fruits (%)	For green fruits (%)	For whole fruits (%)
YOLOv3	97.0	95.2	96.1
Improved YOLOv3	99.4	99.3	99.3

**Figure 10:**
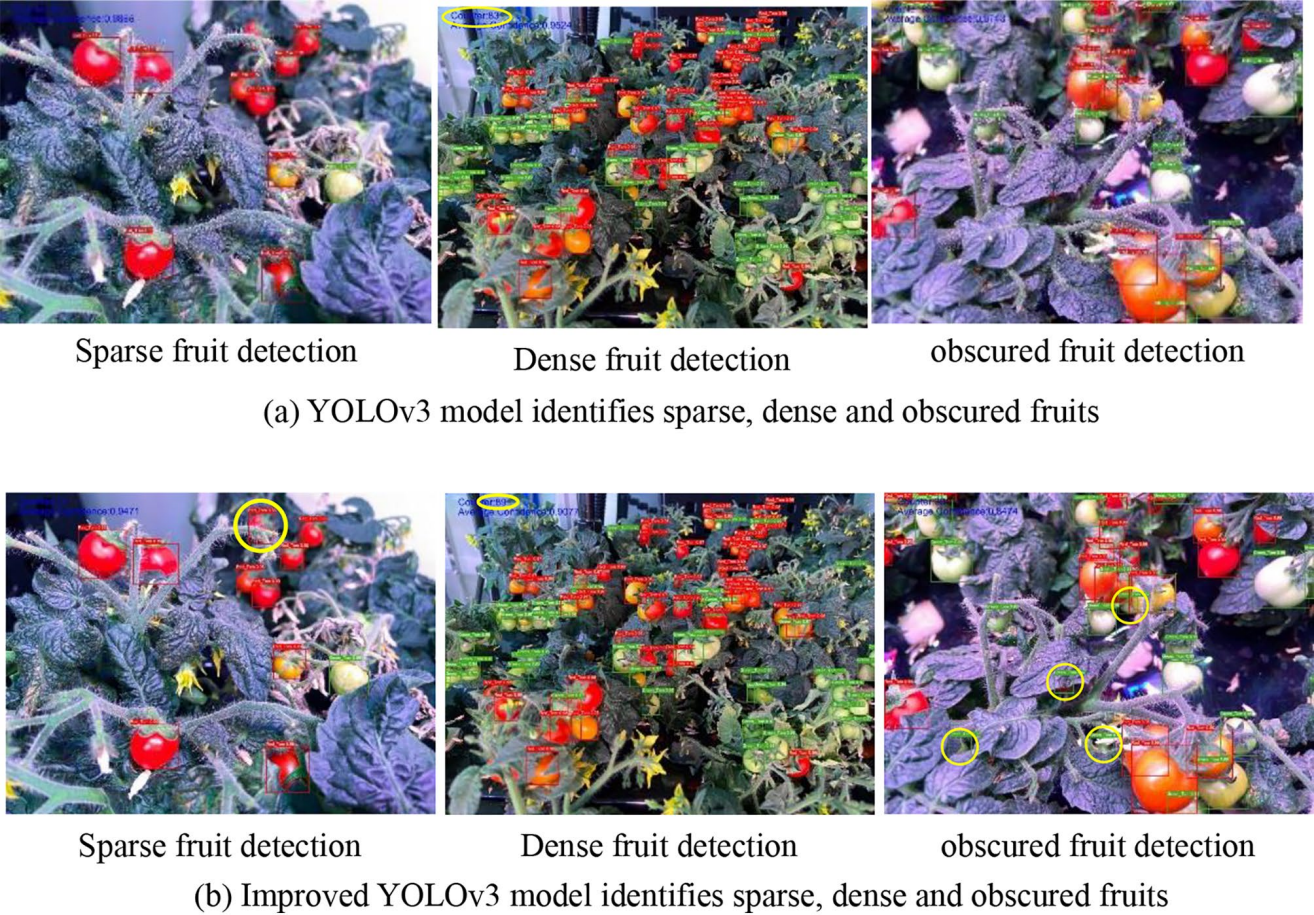
Fruit recognition effect in special field of view.

In addition, in the process of image acquisition, due to the change of the relative posture between the image acquisition system and the tomato plant and the irregularity of fruit growth, the tomato fruit shows sparse, dense and occluded phenomena in the field of view, as shown in Fig. 10. The recognition accuracy of the yield estimation model to the fruit in the special field of view is an important reference to verify the generalization performance of the model.

30 images of sparse fruit, dense fruit and occluded field of view were selected, respectively, and the yield estimation accuracy of the two models was shown in Table 5. The yield estimation accuracy of the improved YOLOv3 model for sparse red fruit and green fruit increased by 2.9% and 2.8% respectively, for dense red fruit and green fruit increased by 5.9% and 7.1% respectively, and for sheltered red fruit and green fruit increased by 9.2% and 9.4% respectively. It can be seen that the improvement of the model can improve the accuracy of tomato yield estimation under three kinds of special field of view, and the effect is more obvious for dense fruit and shaded fruit.

**Table 5:** Yield estimation accuracy of tomato in special field of view.

Model/algorithm	For red fruits			For green fruits		
	Sparse	Dense	Occluded	Sparse	Dense	Occluded
YOLOv3	96.7	93.6	89.7	96.74	92.3	89.3
Improved YOLOv3	99.6	99.5	98.9	99.5	99.4	98.7

Image acquisition method and data set size have a great impact on model training. Theoretically, the more flexible and diverse the angle, light, focal length, sensitivity and exposure time of image data shooting, the greater the amount of data collected and the more data enhancement methods used, the better the effect of model training and the higher the detection accuracy. In this study, the detection performance is significantly improved after expanding the data set. Using yolov3 of the improved DarkNet53 algorithm, the input image is down-sampled 32 times, 16 times, and 8 times, respectively, to obtain different levels of feature maps. Then, through up-sampling and tensor splicing, the feature maps of different levels are fused into feature maps with the same dimension, which improves the accuracy of small target detection. In addition, the algorithm also outputs a multi-scale feature map that improves target detection in the far and near field of view. The experimental results show that the improved yolov3 not only significantly improves the detection accuracy of small targets similar to Micro-Tom fruit, but also significantly improves the detection accuracy of blocked and blurred tomatoes.

## Conclusions

1. In order to meet the needs of tomato planting yield estimation in intelligent plant factories, the tomato fruit recognition method based on improved YOLOv3 model was studied in order to count and estimate tomato fruit yield under natural growth conditions. By improving the position loss function of traditional YOLOv3, a tomato fruit recognition model under natural growth was established. The recognition accuracy of the improved YOLOv3 model is improved, and the mAP value of the final model is 99.3%, which is 2.8% higher than that of the unimproved YOLOv3 model.
2. In order to verify the validity and generalization performance of the recognition model, field tests were carried out. The experimental results show that, compared with the traditional YOLOv3 model, the accuracy of the improved YOLOv3 model for estimating the yield of red fruit, green fruit and the whole tomato has been improved, reaching 99.4%, 99.3% and 99.3%, respectively.
3. The improved YOLOv3 model has a more significant improvement effect and robustness to dense fruit and occluded fruit. The recognition accuracy of dense red fruit and green fruit is 99.5% and 99.4% respectively, and that of occluded red fruit and green fruit is 98.9% and 98.7% respectively. And the average detection time of a single image is 15 ms after the improved algorithm is loaded into the model, which meets the real-time requirements. The results can provide a reference for the estimation of tomato time series yield in plant factories.

Although this study successfully cultivated Micro-Tom tomato in the PFAL, and took the lead in applying target detection model to detect tomato fruit, providing detection technology for dynamic yield estimation and harvesting robot. However, due to the short growth time of tomato fruit in color conversion period, the amount of data collected is too small, which is seriously unbalanced compared with red fruit and green fruit. In this paper, there is no separate data labeling and detection classification for fruits in color conversion period, but only two kinds of target detection for red fruits and green fruits. In addition, due to the complexity of PFAL environment and the particularity of its application, it brings many difficulties and challenges to the accurate detection and yield estimation of tomato fruit. Therefore, in order to improve the detection accuracy and speed in a complex environment and meet the needs of actual production, further research is needed.

## Data Availability

The Micro-Tom tomato dataset we built is fully available and shareable. The datasets used and analysed during the current study are available from the corresponding author on reasonable request.
